# Paeonol attenuates retinopathy in streptozotocin-induced diabetes in rats by regulating the oxidative stress and polyol pathway

**DOI:** 10.3389/fphar.2022.891485

**Published:** 2022-09-07

**Authors:** Kaveri M. Adki, Yogesh A. Kulkarni

**Affiliations:** Shobhaben Pratapbhai Patel School of Pharmacy & Technology Management, SVKM’s NMIMS, Mumbai, India

**Keywords:** paeonol, electroretinography, aldose reductase, lactate dehydrogenase, oxidative stress

## Abstract

The current research work was planned to study the effects of paeonol in the management of diabetic retinopathy. Diabetes was induced in male Sprague Dawley rats using Streptozotocin (55 mg/kg, *i.p.*). After 4 weeks, the diabetic animals were treated with paeonol at a dose of 50, 100, and 200 mg/kg body weight daily for the next 4 weeks. At the end of treatment, retinal physiology was studied by recording an electroretinogram (ERG); biochemical parameters and oxidative stress were estimated. The histopathology of the retina was also carried out at the end of the study. The ERG of paeonol-treated animals showed a significant improvement in a-wave amplitude, b-wave amplitude, a-wave latency, and b-wave latency (*p* < 0.001) at 15 cd s/m^2^ when compared with the diabetic control animals. The paeonol treatment (200 mg/kg) in diabetic animals showed a significant decrease in the plasma glucose level (*p* < 0.001) when compared to the animals in diabetic control group. Paeonol also significantly decreased the lactate dehydrogenase, aldose reductase, and sorbitol dehydrogenase levels when compared with the diabetic control animals. The oxidative stress in the eye was significantly reduced after the paeonol treatment in the diabetic rats. The histopathology showed a significant reduction (*p* < 0.05) in the retinal thickness after the paeonol treatment. The results of the study indicate that paeonol can be considered an effective management option for diabetic retinopathy.

## Introduction

Diabetic retinopathy is one of the major microvascular complications of diabetes. Prolonged uncontrolled hyperglycemia triggers damage to capillary endothelial cells present in the retina (Hammes, 2018). The main causes of diabetic retinopathy include modifiable risk factors (poor glucose control, obesity, dyslipidemia, hypertension, smoking, etc.) and unmodifiable risk factors (family history, age, genetics, type of diabetes, and duration of diabetes). Oxidative stress, inflammation, and metabolic disease are the other risk factors linked to diabetic retinopathy (Congdon et al., 2003). It is predicted that about one in three individuals with diabetes is affected by retinopathy and one in ten can develop permanent vision loss (Yau et al., 2012).

Diabetic retinopathy is mainly characterized by capillary degeneration, basement membrane thickening, increase in vascular permeability, microaneurysm, neovascularization, and intramural pericyte death (Dhoot et al., 2018). The leakage and accumulation of extracellular fluid in the macula of the eye lead to the development of macular edema in diabetics. Macular edema induces the development of new blood vessels in the retina, which may cause visual disturbances and blindness. If not treated during the early stage, diabetic retinopathy may lead to visual disturbance and blindness (Frank, 2004; Forbes and Cooper, 2013). The pathophysiology of diabetic retinopathy is very complex and is not clearly understood. The scientific literature revealed that the significant dilation of retinal blood vessels causes an increase in pericyte damage. Pericytes generally provide structural support to the blood vessels and capillaries. Damage to pericytes may lead to the progression of microaneurysm (Ahsan, 2015). The multiple signaling pathways such as polyol, hexosamine, and protein kinase C are involved in the development of diabetic retinopathy. Stimulation of these pathways may cause the release of inflammatory mediators, reactive oxygen species, some enzymes, and other components (Steele et al., 2008).

Many natural products have been reported for their beneficial effects in various diseases. Paeonol is a phenolic metabolite present in plants like *Dioscorea japonica* var. *Japonica* [Dioscoreaceae], *Paeonia suffruticosa Andrews* [Paeoniaceae], *Arisaema erubescens* (Wall.) Schott [Araceae], and *Paeonia lactiflora* Pall [Paeoniaceae]. Paeonol has been reported for diverse pharmacological activities such as anti-inflammatory, cardioprotective, neuroprotective, nephroprotective, and anti-tumor (Zhang et al., 2019; Adki and Kulkarni, 2020). Liu and coworkers reported the antidiabetic and antioxidant effect of paeonol at a dose of 50 and 100 mg/kg in streptozotocin (STZ)-induced diabetic rats. Paeonol treatment in diabetic rats showed decrease in blood glucose levels, glycosylated serum proteins, and serum advanced glycation end products (AGEs) levels. Study confirmed that paeonol treatment decreased expressions of receptor for advanced glycation end products (RAGE) and nuclear factor kappa B (NF-κB) in diabetic animals. Additionally, paeonol significantly increased glutathione content and remarkably decreased induced nitric oxide synthase activity in diabetic animals via regulating the AGEs/RAGE/NF-κB pathway (Liu et al., 2013).

However, there is no scientific evidence on the role of paeonol in diabetic retinopathy. Hence, the present research work was designed to evaluate the effect of paeonol in diabetic retinopathy in rats.

## Materials and methods

### Chemicals and drug

Streptozotocin (STZ) was purchased from MP Biomedicals (United States). Paeonol, d-fructose, and DL-glyceraldehyde were purchased from Sigma-Aldrich (St. Louis, MO, United States).

### Experimental animals

Male Sprague Dawley rats with a weight of 180–220 g were purchased from the National Institute of Biosciences, Pune, India. All animals were housed in the standard laboratory environment (12 h of light/dark cycle, temperature 22 ± 2°C, and humidity 75 ± 5%) throughout the study. The animals received a standard pellet diet purchased from Nutrivet Life Sciences, India and filtered water *ad libitum* throughout the study. The animal study protocol was reviewed, and approved by the Institutional Animal Ethics Committee [Shri Vile Parle Kelavani Mandal’s animal facility, Mumbai] (protocol approval number CPCSEA/IAEC/P-80/2018). All the experimental procedures on the animals were conducted in compliance with the Committee for the Purpose of Control and Supervision of Experiments on Animals guidelines, Government of India.

### Induction of diabetes and treatment

After 7 days of acclimatization, the animals were weighed and fasted (8 h). The STZ solution was prepared by 0.1 M ice-cold sodium citrate buffer (pH 4.4). Diabetes was induced in the rats with a single intraperitoneal injection of STZ (55 mg/kg) (Garud and Kulkarni, 2017). One week after administration of STZ, blood was withdrawn from the retro orbital plexus of animals and collected in microcentrifuge tubes containing 0.5% disodium ethylenediaminetetraacetate dehydrate (EDTA) salt solution. The blood samples were centrifuged at 5,000 rpm for 10 min by using bench top centrifuge (REMI, India) and the plasma was collected. The plasma glucose levels were estimated by using a diagnostic kit (Transasia Biomedicals Ltd, India) with the help of a biochemical analyzer (ERBA Chem 7, Germany).

One group containing eight animals received 0.5% carboxy methyl cellulose (CMC) by the oral route and this group was considered as the “normal control group.” A total of 32 experimental animals with plasma glucose levels of more than 250 mg/dl were considered diabetic and used for the study. After 4 weeks of diabetes induction, the animals were divided in four groups containing eight in each group. Group 1 containing the diabetic animals was assigned as diabetic control and received 0.5% CMC by oral route. The diabetic animals in groups 2, 3, and 4 were treated with paeonol at a dose of 50, 100, and 200 mg/kg, respectively, by the oral route. All treatments were given for 4 weeks.

### Evaluation parameters

#### Electroretinography

All the animals were housed in the dark for 12 h at the end of the paeonol treatment. The animals were anesthetized using urethane at dose 1,200 mg/kg via an intraperitoneal injection, and the pupils were dilated with 0.5% w/v tropicamide ophthalmic solution. The rat ERG recording electrode (LKC Technologies, MD, United States) was used to record ERG. Each animal was subjected to flashes of light for 10 ms, and ERG was recorded using Powerlab data acquisition system (AD instruments, Australia) (Bhatt and Addepalli, 2010). The ERG was recorded for each animal with pulse intensity 15 cd s/m^2^. The a-wave amplitude, b-wave amplitude, and a-wave and b-wave latency (time-to-peak) were measured.

### Biochemical parameters

After recording ERG, blood was collected from retro-orbital plexus, and the plasma was separated. Glucose and lactate dehydrogenase were estimated by using diagnostic kits (Transasia Bio-medicals Ltd., India). The animals were sacrificed (CO_2_ asphyxiation), and the eyes were isolated carefully and weighed. One eye was preserved in modified Davidson’s fixative solution for histopathology study. Lens homogenate was used for the determination of biochemical assay.

### Preparation of lens homogenate and protein estimation

The lens was separated from the eyeball and subjected to homogenization in a phosphate buffer (pH 7.4) using a probe homogenizer (Polytron PT 2500E, Kinematica, Switzerland). Homogenates were centrifuged using a cold centrifuge (Eppendorf, Germany) at 2,500 rpm. The supernatant was used for the determination of protein concentration and aldose reductase assay. The protein estimation was carried out by using Lowry’s method (Lowry et al., 1951). The lens homogenates were diluted 20 times with millipore water and used for protein estimation. The bovine serum albumin (BSA) was used as standard and millipore water as blank. 10 µl of tissue homogenate and 10 µL alkaline copper sulfate were added to 96 well plates and incubated for 10 min at room temperature. 0.1 ml of Folin’s phenol reagent was added, mixed thoroughly, and incubated at 37°C for 10 min. The absorbance was recorded at 640 nm against blank in a microplate spectrophotometer (Epoch 2, BioTek, United States). The standard curve of BSA was used to determine the protein concentration present in the lens homogenate and expressed in mg/ml.

### Determination of aldose reductase activity

The assay was carried out by using 0.067 M phosphate buffer (70 µl), 25 × 10^–5^ M NADPH (10 µl), 5 × 10^–4^ M DL-glyceraldehyde (10 µl) and lens supernatant (10 µl). Wells containing all components except DL-glyceraldehyde were considered blank. The absorbance was recorded against blank at 340 nm and 60 s time interval for 3 min using a microplate spectrophotometer (Epoch 2, BioTek, United States). The aldose reductase activity was calculated ΔOD/min/mg protein and expressed as U/mg protein (Patel et al., 2012).

### Determination of sorbitol dehydrogenase activity

Sorbitol dehydrogenase assay was carried out according to methods described previously by Lindstad with slight modification. 117.5 μL of 100 mM Triethanolamine Buffer (pH 7.6), 250 µL 1.1 M d-Fructose Solution, and 50 µL 12.8 mM *β*-nicotinamide adenine dinucleotide (reduced form) solution were added to the blank plate. All solutions were mixed by inversion and the absorbance monitored at 340 nm (until it gives constant absorbance) using a temperature-controlled microplate spectrophotometer (Epoch 2, BioTek, United States) at 25°C. Then 50 μL tissue homogenate was added and mixed immediately. Absorbance was recorded at 340 nm for 3 min and the absorbance/minute was obtained wusing the maximum linear rate. 1% w/v BSA solution was used as blank instead of homogenate (Lindstad et al., 1992). Sorbitol dehydrogenase activity was calculated and expressed in U/mg protein using the following formula:

$$\text{Units}/\mu \text{L}\;\text{homogenate}=\frac{\left(\Delta \text{At}-\Delta \text{Ab}\right)\;\left(\text{total}\;\text{volume}\right)\;\left(\text{df}\right)}{\;6.22\left(\text{volume}\;\text{of}\;\text{homogenate}\right)},$$

$$\text{Units}/\mathrm{mg}\;\mathrm{protine}=\frac{\text{Units}/\mathrm{mL}\;\text{homogenate}}{\;\text{mg}/\mathrm{mL}\;\mathrm{protine}\;\mathrm{in}\;\mathrm{the}\;\text{homogenate}}.$$

Here, ΔAt is a change in absorbance/min of test, ΔAb is a change in absorbance/min of blank, df is dilution factor, and 6.22 is the micromolar extinction coefficient of *β*-NADH at λmax 340 nm.

### Estimation of oxidative stress parameter

The lens homogenate (10%) was used for the estimation of glutathione (GSH) and malondialdehyde (MDA). The homogenate was subjected to centrifugation (Eppendorf, Germany) at 2,500 g for 10 min at 4°C to get the post-nuclear supernatant which was used for the estimation of catalase activity. Further, the homogenate was centrifuged at 10,000 rpm for 20 min at 4°C to obtain the post-mitochondrial supernatant which was used for the estimation of superoxide dismutase (SOD) activity. The MDA assay was performed as described by Ohkawa (Ohkawa et al., 1979), GSH as described by Ellman (Ellman, 1959), SOD as described by Paoletti and Mocali (Paoletti et al., 1990), and catalase as described by Luck (Luck, 1965).

### Histopathology

All the sections of the retina were stained by Mayer’s Hematoxylin & Eosin and observed under a digital microscope with ×400 magnification (Kjellström et al., 2006).

### Statistical data analysis

The statistical data was assessed by using GraphPad Prism five software. Significant differences between the normal control, diabetic control, and paeonol treatment groups were evaluated by analysis of variance (ANOVA) test followed by Bonferroni compared selected pairs of column tests. All the data were expressed in Mean ± SD. *p* values less than 0.05 were considered as significant.

## Results

### Effect of paeonol on electroretinography

**FIGURE 1:**
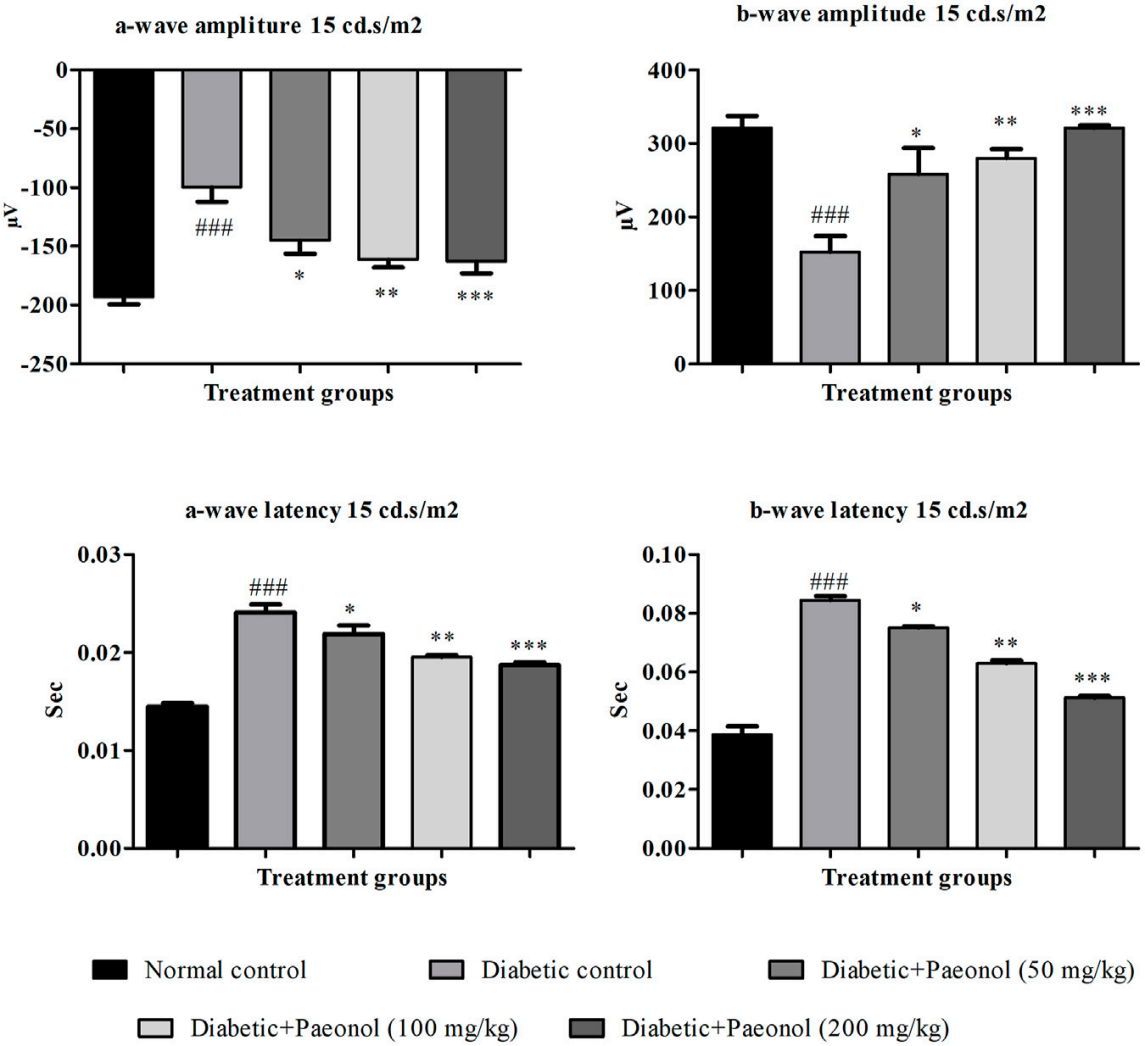
Effect of paeonol on electroretinography at 15 cd s/m^2^. All values are expressed as Mean ± SD (n = 8). ^###^ *p* < 0.001 when compared with the normal control group and ^*^ *p* < 0.05, ^**^ *p* < 0.01, ^***^ *p* < 0.001 when compared with the diabetic control.

Electroretinography was performed to evaluate the electrical response of the eye’s light-sensitive rod and cone cells. The paeonol-treated diabetic animals showed improvement in ERG at 15 cd s/m^2^ when compared to the diabetic control animals (Figure 1).

### a-Wave amplitude

The experimental animals in the diabetic control group showed a significant increase in the a-wave amplitude when compared to the normal animals (*p* < 0.001). The paeonol treatment at a dose of 50, 100, and 200 mg/kg significantly decreased (*p* < 0.05, *p* < 0.01, and *p* < 0.001 respectively) the a-wave amplitude in the experimental animals when compared to the animals in the diabetic control group.

### b-Wave amplitude

The rats in the diabetic control group showed a significant decrease (*p* < 0.001) in the b-wave amplitude when compared to the normal animals. The paeonol treatment at a dose of 50, 100, and 200 mg/kg significantly increased (*p* < 0.05, *p* < 0.01, and *p* < 0.001) the b-wave amplitude in the experimental animals when compared to the animals in the diabetic control group.

### a-Wave latency

The rats in the diabetic control group showed a significant increase (*p* < 0.001) in the a-wave latency when compared to the normal animals. The paeonol treatment at a dose of 50, 100, and 200 mg/kg significantly decreased (*p* < 0.05, *p* < 0.01, and *p* < 0.001, respectively) the a-wave latency in the experimental animals when compared to the animals in the diabetic control group**.**

### b-Wave latency

The rats in the diabetic control group showed a significant increase (*p* < 0.001) in the b-wave latency when compared to the normal animals. The paeonol treatment at a dose of 50, 100, and 200 mg/kg significantly decreased (*p* < 0.05, *p* < 0.01, and *p* < 0.001) the b-wave latency in the experimental animals when compared to the animals in the diabetic control group.

### Biochemical parameters

The diabetic rats showed a significant increase in the plasma glucose and lactate dehydrogenase enzyme (*p* < 0.001, *p* < 0.01) when compared to the normal animals. The diabetic animals treated with paeonol at a dose of 100 and 200 mg/kg showed a significant decrease in plasma glucose levels (*p* < 0.01 and *p* < 0.001, respectively) when compared to the animals in the diabetic control group. The paeonol treatment at a dose of 200 mg/kg in the diabetic rats decreased the lactate dehydrogenase enzyme levels significantly (*p* < 0.05) when compared to the animals in the diabetic control group.

The experimental animals in the diabetic control group showed a significant increase in the aldose reductase and sorbitol dehydrogenase enzyme levels (*p* < 0.01, *p* < 0.001) when compared to the animals in the normal control group. The paeonol treatment at a dose of 100 and 200 mg/kg significantly decreased the aldose reductase level (*p* < 0.05, *p* < 0.01) when compared to the animals in the diabetic control group. The paeonol treatment at all selected doses significantly decreased the sorbitol dehydrogenase levels when compared to the animals in the diabetic control group (Table 1).

**TABLE 1:** Effect of paeonol on biochemical parameters.

Parameter	Plasma glucose (mg/dl)	Plasma lactate dehydrogenase (U/l)	Aldose reductase (U/mg of protein)	Sorbitol dehydrogenase (U/mg of protein)
Group				
Normal control	96.85 ± 6.88	165.8 ± 51.49	0.8468 ± 0.70	0.03905 ± 0.02
Diabetic control	507.6 ± 133.7^###^	435.6 ± 182.2^##^	5.032 ± 2.37^##^	0.1430 ± 0.04^###^
Diabetic + paeonol (50 mg/kg)	423.1 ± 121.7	317.9 ± 114.3	2.502 ± 1.94	0.09206 ± 0.04^**^
Diabetic + paeonol (100 mg/kg)	345.0 ± 90.55^**^	290.8 ± 80.5	1.717 ± 1.25^*^	0.06616 ± 0.02^***^
Diabetic + paeonol (200 mg/kg)	251.0 ± 81.55^***^	198.5 ± 107.47^*^	0.9787 ± 0.43^**^	0.05986 ± 0.02^***^

All values are expressed as Mean ± SD (n = 8). ^###^ *p* < 0.001, ^##^ *p* < 0.01 when compared with the normal control group and ^*^ *p* < 0.05, ^**^ *p* < 0.01, ^***^ *p* < 0.001 when compared with the diabetic control.

### Oxidative stress parameters

The diabetic rats showed a significant decrease in GSH level (*p* < 0.001) when compared to the normal animals. The paeonol treatment at a dose of 200 mg/kg significantly increased (*p* < 0.05) the GSH level in the diabetic rats. The diabetic rats showed a significant increase in the MDA level (*p* < 0.01) when compared to the normal animals. The paeonol treatment at a dose of 200 mg/kg significantly decreased (*p* < 0.05) the MDA level in the diabetic rats. The diabetic rats showed a significant decrease in the SOD (*p* < 0.01) and catalase (*p* < 0.05) level when compared to the normal animals. The paeonol treatment at a dose of 200 mg/kg significantly increased the SOD (*p* < 0.05) and catalase (*p* < 0.01) level in the diabetic rats (Table 2).

**TABLE 2:** Effect of paeonol on the oxidative stress parameters in the lens of experimental rats.

Parameter	GSH (µM/mg of protein)	MDA (µM/mg of protein)	SOD (U/mg of protein)	Catalase (µM of H_2_O_2_/mg of protein)
Group				
Normal control	5.898 ± 2.31	5.32 ± 1.5	0.0518 ± 0.02	0.01717 ± 0.012
Diabetic control	1.980 ± 0.92^###^	10.36 ± 6.37^##^	0.0178 ± 0.005^##^	0.00077 ± 0.0006#
Diabetic + paeonol (50 mg/kg)	2.595 ± 1.13	7.943 ± 1.05	0.0293 ± 0.009	0.003272 ± 0.001
Diabetic + paeonol (100 mg/kg)	3.383 ± 2.76	6.587 ± 1.86	0.033 ± 0.02	0.00834 ± 0.007*
Diabetic + paeonol (200 mg/kg)	4.764 ± 1.33^*^	6.244 ± 2.19^*^	0.0483 ± 0.02^*^	0.01415 ± 0.011**

All values are expressed as Mean ± SD (n = 8). ^###^ *p* < 0.001, ^##^ *p* < 0.01, ^#^ *p* < 0.05 when compared to the normal control group and ^*^ *p* < 0.05, ^**^ *p* < 0.01 when compared with the diabetic control.

### Histopathology of retina

**FIGURE 2:**
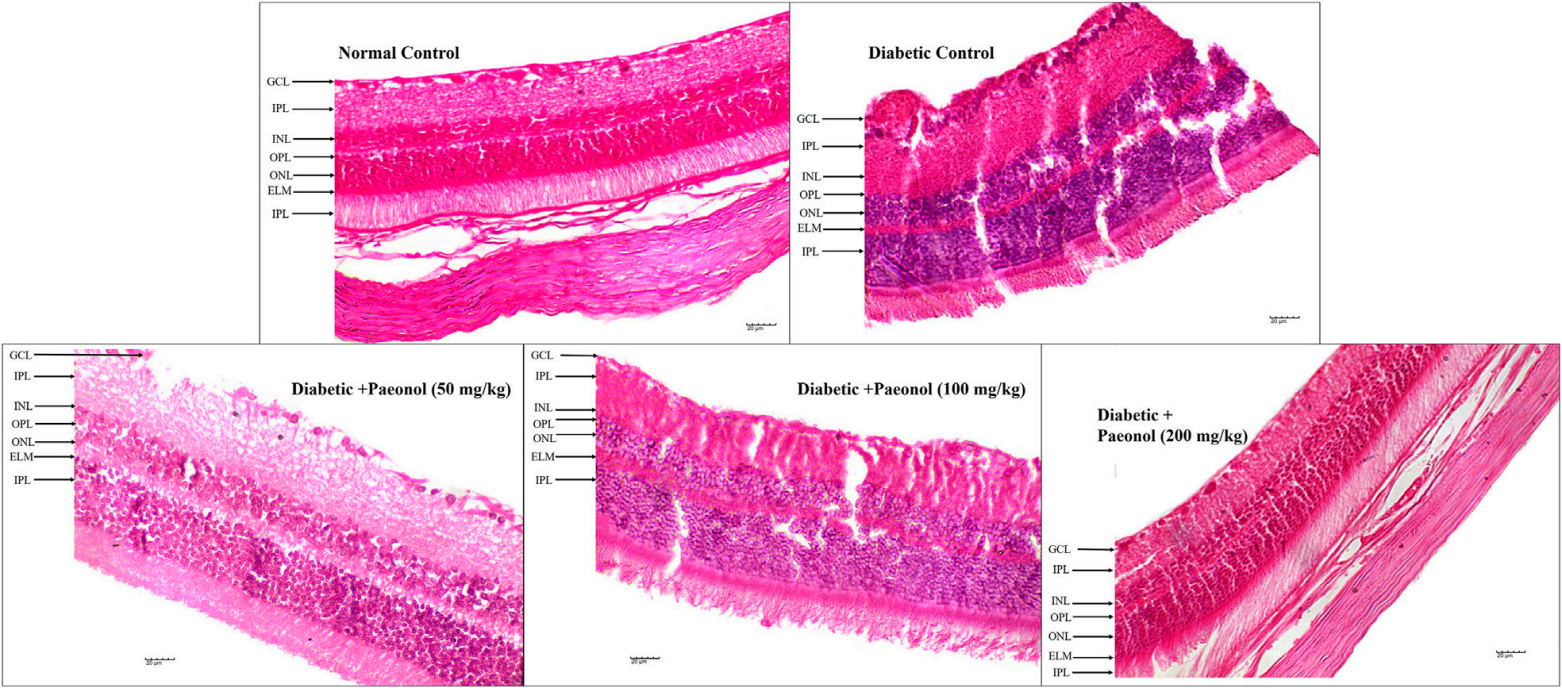
Effect of paeonol on the histopathology of the eye in the experimental animals (400 X) (GCL, ganglion cell layer; IPL, inner plexiform layer; INL, inner nuclear layer; OPL, outer plexiform layer; ONL, outer nuclear layer, ELM, external limiting membrane).

The histopathology of the diabetic rats showed a significant increase in the thickness of the retina (*p* < 0.001) when compared to the normal animals. The paeonol treatment at a dose of 200 mg/kg significantly decreased (*p* < 0.05) the retinal thickness in the diabetic rats (Figure 2; Table 3).

**TABLE 3:** Effect of paeonol on the retinal thickness of experimental rats.

Group	Normal control	Diabetic control	Diabetic + paeonol (50 mg/kg)	Diabetic + paeonol (100 mg/kg)	Diabetic + paeonol (200 mg/kg)
Details					
Thickness of retina (µm)	81.29 ± 1.61	121.52 ± 10.59^###^	110.72 ± 13.07	105.86 ± 8.93	96.61 ± 11.33^*^

All values are expressed as Mean ± SD ^###^ *p* < 0.001 when compared with the normal control group and ^*^ *p* < 0.05 when compared with the diabetic control.

## Discussion

The literature search was done for a dose selection of paeonol. The literature reported that the paeonol showed an antidiabetic and antioxidant effect at a dose of 50 and 100 mg/kg in streptozotocin (STZ)-induced diabetic rats (Liu et al., 2013). The maximally tolerated dose of paeonol was reported as 5,000 mg/kg in an acute toxicity study in rats. Paeonol was found to be safe at a dose of 50, 100, and 200 mg/kg in a repeated dose toxicity study (Adki et al., 2020). Hence, in the current study we used a 50, 100, and 200 mg/kg dose of paeonol to study the effect in diabetic retinopathy.

The ERG was carried out by measuring the functional response photoreceptors, and downstream retinal cells in experimental animals to a light stimulus that builds on a previously published approach (Joseph Phillips et al., 2010). In the current study, the ERG of the diabetic control group showed a loss of amplitude or sometimes increased amplitude of a-wave and b-wave. The paeonol-treated animals showed an improvement in the ERG when compared to the diabetic control. The ERG results also confirm that paeonol has the potential to protect the retinal damage or dystrophies occurring due to oxidative stress, extravasation of tissue, and retinal leakage.

The lactate dehydrogenase enzyme has the ability to catalyze lactate to pyruvate in the presence of nicotinamide adenine dinucleotide (NAD+). When cells are injured or damaged, the lactate dehydrogenase enzyme is released into the blood (Markert, 1984). The results showed that the paeonol-treated group significantly decreased the plasma lactate dehydrogenase levels compared to the diabetic control. Hence, the aldose reductase enzyme activity was carried out in the experimental animals. The results showed that paeonol treatment inhibited the aldose reductase activity when compared to the diabetic control. These results are in accordance with the previously reported literature (Liu et al., 2019).

Paeonol reduced oxidative stress due to diabetes and diabetic complications such as diabetic encephalopathy (Min et al., 2007). In the current study too, the oxidative stress of the eye was also found to decrease in the paeonol treatment group by enhancing the levels of GSH, SOD, and catalase and declining the levels of MDA when compared to the diabetic control.

The histopathology study showed a decrease in retinal damage in the paeonol-treated animals.

## Conclusion

Paeonol was found to be effective in diabetic retinopathy in rats at a high dose used in the study. From the results of the study, it can be concluded that paeonol can be used for the management of diabetic retinopathy.

## Data Availability

The raw data supporting the conclusion of this article will be made available by the authors, without undue reservation.
